# Bacteriophage-derived endolysins as innovative antimicrobials against bovine mastitis-causing streptococci and staphylococci: a state-of-the-art review

**DOI:** 10.1186/s13028-024-00740-2

**Published:** 2024-05-20

**Authors:** Niels Vander Elst

**Affiliations:** Department of Neuroscience, Karolinska Institutet, Biomedicum 7D, Solnavägen 9, 17165 Solna, Stockholm, Sweden

**Keywords:** Bacteriophage-derived endolysins, Bovine mastitis, Dairy industry, Endolysins, Gram-positive, Homology-based, *Staphylococcus*, *Streptococcus*, Veterinary medicine

## Abstract

Bacteriophage-encoded endolysins, peptidoglycan hydrolases breaking down the Gram-positive bacterial cell wall, represent a groundbreaking class of novel antimicrobials to revolutionize the veterinary medicine field. Wild-type endolysins exhibit a modular structure, consisting of enzymatically active and cell wall-binding domains, that enable genetic engineering strategies for the creation of chimeric fusion proteins or so-called ‘engineered endolysins’. This biotechnological approach has yielded variants with modified lytic spectrums, introducing new possibilities in antimicrobial development. However, the discovery of highly similar endolysins by different groups has occasionally resulted in the assignment of different names that complicate a straightforward comparison. The aim of this review was to perform a homology-based comparison of the wild-type and engineered endolysins that have been characterized in the context of bovine mastitis-causing streptococci and staphylococci, grouping homologous endolysins with ≥ 95.0% protein sequence similarity. Literature is explored by homologous groups for the wild-type endolysins, followed by a chronological examination of engineered endolysins according to their year of publication. This review concludes that the wild-type endolysins encountered persistent challenges in raw milk and in vivo settings, causing a notable shift in the field towards the engineering of endolysins. Lead candidates that display robust lytic activity are nowadays selected from screening assays that are performed under these challenging conditions, often utilizing advanced high-throughput protein engineering methods. Overall, these recent advancements suggest that endolysins will integrate into the antibiotic arsenal over the next decade, thereby innovating antimicrobial treatment against bovine mastitis-causing streptococci and staphylococci.

## Background

### Endolysins are bacteriophage-encoded peptidoglycan hydrolases

**Fig. 1:**
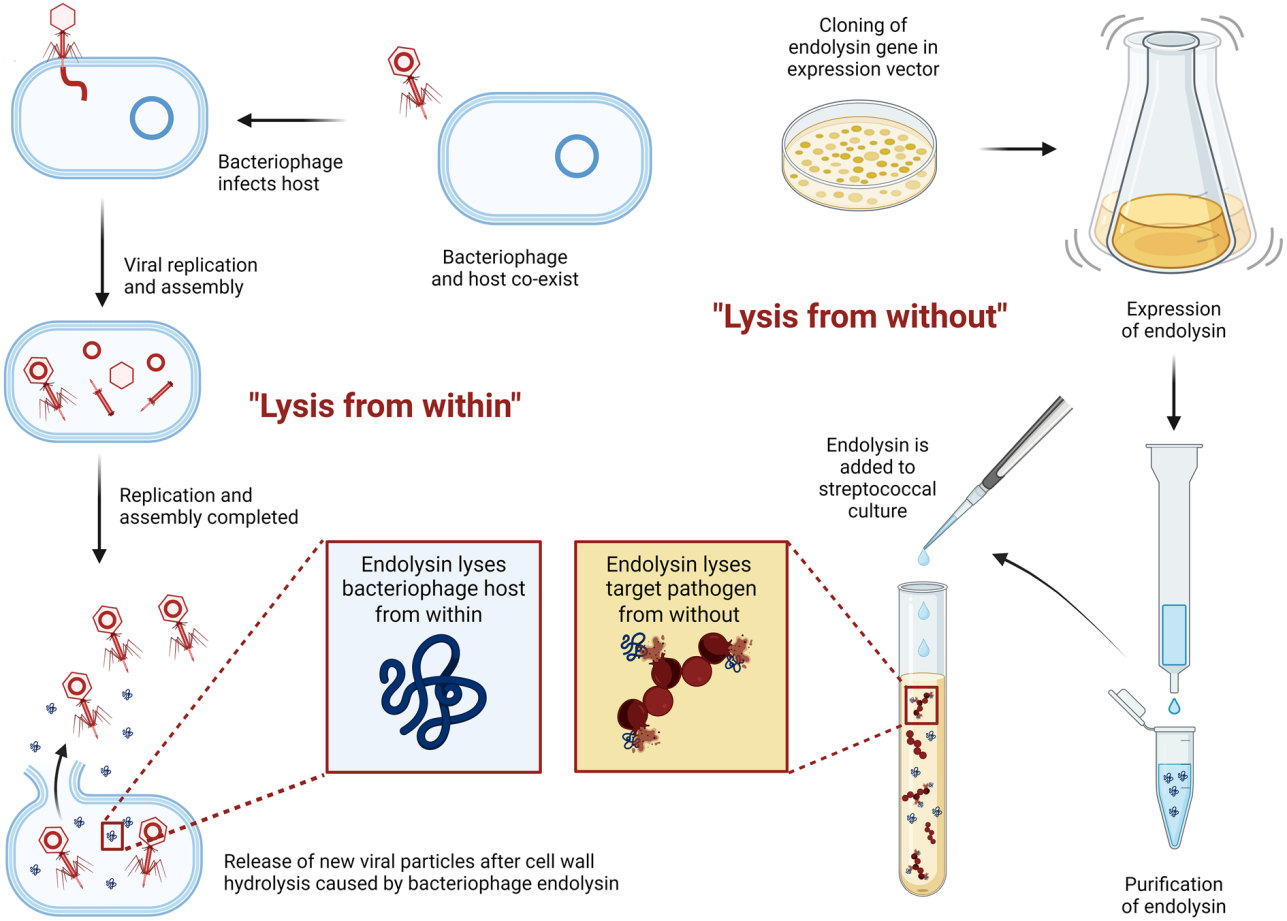
The action of endolysins in a ‘lysis-from-within’ versus ‘lysis-from-without’ scenario. Bacteriophages employ endolysins at the end of their lytic replication cycle to release newly assembled viral particles from the infected host, causing ‘lysis-from-within’. In the “lysis-from-without” scenario, the endolysin gene is cloned in a vector, after which the endolysin is expressed and purified. In the case of Gram-positive pathogens, the purified endolysin retains its functionality when applied externally to the targeted pathogen (created with https://biorender.com/)

Bacteriophages, or shortly phages, are viruses that infect bacteria [1–3]. Phages recognize specific receptors on the cell wall of bacterial species or strains, which cause them to adsorb and inject their DNA. A successful phage infection subsequently can result in two different outcomes: (i) the phage enters a lysogenic state, in which its genome is inserted into the bacterial genome and replicates together with the bacterium (*i.e.*, prophage), or (ii) the phage passes a lytic cycle in which it reprograms the bacterial host cell to synthesize and assemble new viral particles [2, 3]. These lysogenic and lytic cycles can transfer into one another, but strictly virulent phages exist and replicate by a lytic infection cycle only. At the end of the lytic cycle, endolysins are produced by the phage to release these newly assembled viral particles from the infected host. This process is known as ‘lysis-from-within’ (Fig. 1) [4]. In this process, a secondary phage protein named a holin will first perforate the lipid layer of the inner bacterial membrane, which then enables endolysins to hydrolyse the outer peptidoglycan layer [5]. Thus, endolysins are peptidoglycan hydrolases that degrade the bacterial cell wall. In the case of Gram-positive bacteria, endolysins are known to retain this functionality when they are recombinantly produced in the laboratory and incubated with Gram-positive bacteria, which is a process referred to as ‘lysis-from-without’ (Fig. 1) [6]. Indeed, when endolysins hydrolyse the bacterial cell wall of Gram-positive bacteria, the high internal osmotic pressure inside the bacterial cell causes the bacterium to ‘burst’ or ‘lyse’. From that perspective, endolysins have been proposed and investigated as promising, novel antimicrobials [3, 4, 7, 8].

### Endolysins are modular proteins amenable to genetic engineering

Endolysins derived from Gram-positive bacteriophages typically feature a modular structure [4, 7, 9]. They consist of one or more so-called enzymatically active domains [EADs; introduced after (i)] and cell wall-binding domains [CBDs; introduced after (ii)], which confer either peptidoglycan hydrolysis or binding activity, respectively. EADs are sometimes also referred to as catalytic domains. These domains are usually, but not exclusively, coupled by proline- or lysine-rich regions referred to as linkers [introduced after (iii)] [10, 11]. This modular structure of endolysins allows the easy generation of chimeric fusion proteins or so-called ‘engineered endolysins’ [4, 7, 9]. Indeed, the EADs or CBDs of an endolysin can readily be altered by making changes on DNA level (e.g., PCR followed by restriction/ligation in a vector and overexpression in a host). This biotechnological strategy to engineer endolysins has been extensively pursued and endolysins that combine EADs and CBDs of different origin have been created after this concept was introduced to the field. Many different types of EADs and CBDs have been described, which are categorized based on the specific position where they either hydrolyze the peptidoglycan or bind the cell wall, respectively.

**Fig. 2:**
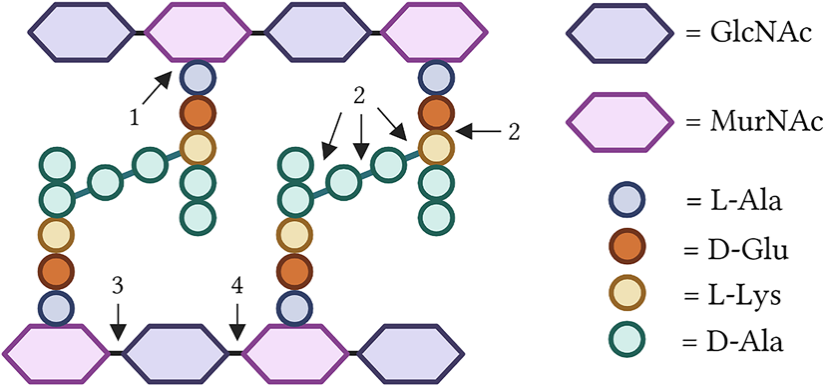
Schematic representation of the streptococcal bacterial peptidoglycan structure, including the endolysin cleavage sites. The peptidoglycan is composed of repeating sugar units, n-acetyl-glucosamine (GlcNAc) and n-acetyl-muramic acid (MurNAc), which are cross-linked via a d-Alanine (d-Ala) interpeptide bridge between l-Lysine (l-Lys) and d-Ala residues. The chains also contain l-Ala and d-glutamic acid (d-Glu). The cleaved bonds and major classifications of enzymatically active domains (EADs) of endolysins are indicated: (1) n-acetyl-muramoyl-l-ala amidase; (2) various endopeptidases; (3) n-acetyl-β-d-muramidase; (4) n-acetyl-β-d-glucosaminidase (created with https://biorender.com/)

(i) EADs are biochemically categorized based on the peptidoglycan bond(s) that they hydrolyze (Fig. 2) [4, 7, 9]. The most common types of EADs found in endolysins from Gram-positive bacteriophages, are: (1) n-acetyl-muramoyl-l-alanine (ala) amidases (subtypes 2, 3 and 5), (2) endopeptidases such as M23 peptidases or cysteine/histidine-dependent aminohydrolases/peptidases (CHAPs), (3) n-acetyl-β-d-muramidases, and (4) n-acetyl-β-d-glucosaminidases [12–14]. Amidases hydrolyse the bond between the n-acetyl-muramic acid (MurNAc) and the first l-ala in the stem peptide, whereas endopeptidases can impact different bonds in the stem peptide, or the interpeptide bridge, or between those two latter. Muramidases and glucosaminidases hydrolyze bonds between the MurNAc and n-acetyl-d-glucosamine (GlcNAc) repeating sugar units.
(ii) CBDs recognize certain patterns in and bind to the bacterial peptidoglycan, (lipo)teichoic acids or other cell wall components [4, 7, 9]. They frequently consist of repeated sequences [12]. Multiple studies have shown the binding activity of these CBDs by either fusing them to green fluorescent protein (GFP) or by conjugation of the CBD to a fluorophore (e.g., Alexa Fluor) [15, 16]. In general, an endolysin requires at least one CBD to be functional for Gram-positive endolysins, as was shown by deleting the CBD [15–17]. In most cases, this resulted in loss or inferior activity of the remaining EAD(s). An exception has been described for PlyL and PlyK (a.k.a. LysK) [18, 19]. On the other hand, it is also hypothesized that CBDs play a major role in countering resistance development [20]. Indeed, if endolysins were massively released from the infected host at the end of the lytic cycle, exposure of neighboring Gram-positive bacteria to the endolysins may select for resistant variants in the bacterial population. It is therefore accepted that the CBD plays a major role in keeping the endolysin bound to the lysed host cell. As such, removal of the CBD may in some cases counterintuitively improve the activity of the endolysin, as it can increase the enzyme’s turnover by not keeping it bound to the peptidoglycan of only one bacterial cell [20]. In addition, the iso-electric point (pI) of the endolysin is altered, which may result in an improved affinity for the bacterial cell wall [18, 21]. CBDs are also categorized, but the exact binding place of the peptidoglycan is often not yet fully elucidated and remains understudied. It is reported that LysM recognizes the GlcNAc moiety, whereas SH3 domains interact with both the stem peptide and the interpeptide bridge, more specifically—but not exclusively—the pentaglycine bridge in staphylococcal peptidoglycan [22–24]. CW_7 is believed to bind MurNAc and the region of the adjacent stem peptide [25].
(iii) Linkers, usually composed of proline- or lysine-rich regions, connect the modular domains of an endolysin. These linkers may exhibit diverse properties, such as being short or long, rigid or flexible, or others. The linker regions impact the lytic activity of the endolysin, and this can be improved by selectively editing the linker regions only [10, 11].

Of note, recent insights have revealed that many endolysins form multimers [26, 27]. This is specifically stated to frequently occur in streptococcal endolysins. Multimeric endolysins either consist of multimers of the same endolysin, but endolysin genes frequently contain additional ribosomal binding sites where shorter sequences of the endolysin are translated [26]. An example of a multimeric endolysin is PlyC, which is composed from two separate gene products [27]. The CBD of PlyC self-assembles into a circular octamer to which the EAD is subsequently linked by salt bridges and/or Van der Waals forces.

### In vitro assays to evaluate endolysin activity

**Fig. 3:**
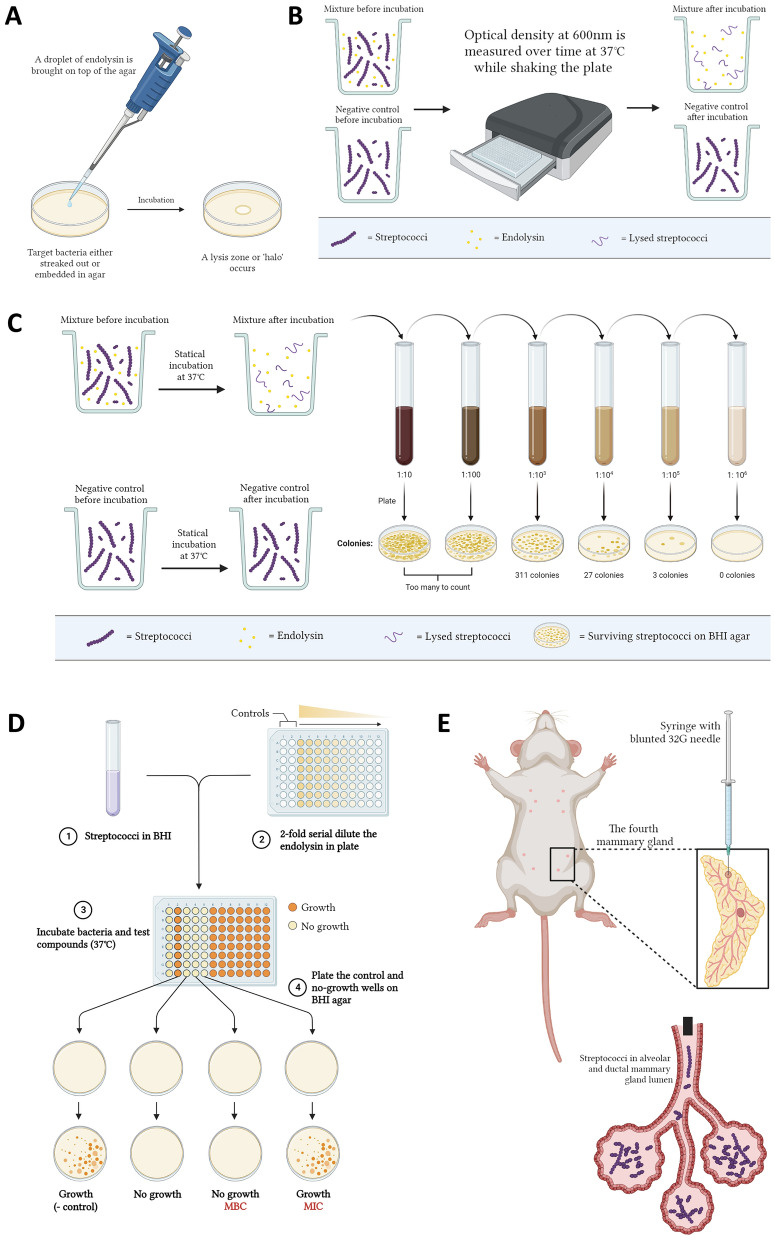
Assays in place to determine the lytic activity of endolysins. **A** In a spot-on-plate or -lawn assay, a droplet of endolysin is brought on top of an agar that contains the target bacteria, after which a lysis zone or ‘halo’ becomes visible. **B** The turbidity reduction assay measures the OD_600nm_ over time to observe a reduction in the turbidity of the target bacteria, which correlates with hydrolysis of the peptidoglycan and osmotic lysis caused by the endolysin. **C** In a time kill assay, triplicates of the target bacteria are incubated at approximately 10^6^ CFU/mL with either an endolysin or a negative control, after which they are serially diluted and plated on an agar. After incubation of the plate at 37 °C during 18 h, CFUs are counted, and the bacterial killing is calculated in comparison with the negative control (difference expressed as Δlog_10_). **D** The target bacteria are challenged with decreasing concentrations of the endolysin in broth and incubated at 37 °C during 18 h. The lowest concentration at which no visible growth in liquid broth is observed, is defined as the minimal inhibitory concentration (MIC). The conditions that show no growth are subsequently plated on an agar to determine if surviving bacteria are present. The lowest concentration at which no surviving bacteria are observed is defined as the minimal bacteriocidic concentration (MBC). **E** In the mouse mastitis model, bovine mastitis-causing streptococci (or other pathogens) are inoculated in the fourth mammary gland pair with a blunted 32G paediatric needle while the mice are under general anaesthesia (created with https://biorender.com/)

The in vitro assays to evaluate endolysin activity constitute the spot-on-plate and -lawn assay, the zymogram, the turbidity reduction assay, the time kill assay and the determination of the minimal inhibitory (MIC) and minimal bacteriocidic (MBC) concentration (Fig. 3).

In a spot-on-plate assay, a drop of endolysin is placed on top of a nutrient-poor agar in which the bacterial target is embedded and incubated at 37 °C (Fig. 3A). If the endolysin is active, a clearing zone or ‘halo’ will become visible [9]. A similar assay is the spot-on-lawn, in which the bacteria are plated on top of a nutrient-rich agar together with a drop of endolysin [28, 29]. If the bacteria are killed by the endolysin, they will not grow, and a halo will become visible. The spot-on-plate assay is regarded more stringent than the spot-on-lawn assay. Of note, the diameter of the halo does not correlate with the lytic activity of the endolysin [4, 9, 30, 31]. It is rather dependent on the size of the endolysin that diffuses through the agar. Indeed, endolysins with a low molecular weight are believed to cause a bigger halo than those with a high molecular weight. The zymogram is not further discussed, as it is not relevant for the reviewed literature in this work, but can be consulted in O’Flaherty et al. [29]. Overall, the spot-on-plate, spot-on-lawn and zymogram assays are highly sensitive to detect enzymatic endolysin activity, as there is a long contact time between the endolysin and the bacteria which are statically embedded in an agar or gel [9]. Therefore, they are regarded qualitative rather than quantitative assays [16].

The turbidity reduction assay (TRA) is a method to evaluate the biochemical activity of an enzyme (Fig. 3B) [16, 31–37]. It measures a decrease of the optical density (OD) over time using a spectrophotometer, typically monitored at a wavelength of 600 nm (OD_600nm_). The TRA measures the hydrolysis of peptidoglycan caused by the endolysin, which results in osmotic lysis and killing of the bacteria [16]. It is a very powerful and useful assay to quantify the biochemical activity of an endolysin compared to a negative control at the end of the TRA (i.e., ΔOD_600nm_), or to calculate the rate at which the bacterial cells are lysed [i.e., (ΔOD_600nm_/min)/µM] [33]. It is also important to note that bacteria at early and mid-exponential phases are much more susceptible than those in the late exponential or stationary phase. For instance, one study showed that the turbidity of a solution of bacteria in the exponential phase decreased by 50% in 15 min, whereas cells harvested in the stationary phase decreased by only 5% at equimolar endolysin concentrations [37].

To quantify bacterial killing caused by the endolysin, a time killing assay (TKA) is more appropriate (Fig. 3C). A TKA challenges a certain number of target bacteria, typically lower than the TRA, during a predetermined time interval [16, 31, 34, 35]. As a standard for endolysins, 10^6^ colony forming units per milliliter (CFU/mL) of target bacteria are usually challenged during a fixed time interval [16, 31]. Thereafter, the bacteria are serially diluted and plated on an agar. This agar is then incubated overnight at the appropriate temperature to determine the surviving CFU/mL. The number of killed bacteria is calculated in comparison with a negative control, typically expressed logarithmically (i.e., Δlog_10_). In the context of mastitis research, a kinetic TKA in both ultra-high temperature treated (UHT) whole milk as well as in (mastitic) raw cow’s milk can also be performed [31, 38, 39]. Note that a milk test setting cannot be used for a TRA given its turbidity.

Alternative antibacterial assays that can be performed to evaluate the antibacterial activity of an endolysin, are the determination of the MIC or MBC (Fig. 3D) [40, 41]. The MIC is defined as the lowest concentration of an antibacterial agent which, under strictly controlled in vitro conditions, completely prevents visible growth of the target bacteria. A MIC assay thus differs from a TKA as it measures the capacity to keep a low number of bacteria under control and to inhibit their growth, whereas a TKA effectively quantifies cell number reduction. To perform a MIC assay, the target bacteria are incubated in broth with decreasing concentrations of the antimicrobial and incubated at 37 °C. To subsequently determine the MBC thereof, the contents of the wells that visibly fully inhibit the growth are plated on an agar. This whole is then incubated and checked for bacterial growth. The MBC is defined as the lowest concentration of an antibacterial agent required to kill all bacteria over a certain period. When the MBC ≤ 4 × MIC, the antibacterial is called bactericidal.

To bridge the gap between in vitro characterization of endolysin activity and subsequent validation in (pre)clinical models, cell culture models have been employed as a useful tool. Furthermore, cell cultures have specifically been used in the context of endolysins to: (i) study cytotoxicity (e.g., PlyC on bovine polymorphonuclear leukocytes (PMN) [42]), and (ii) evaluate intracellular killing (e.g., engineered endolysins carrying so-called cell penetrating peptides (CPPs) [30, 31, 39, 43]). Three major bovine mammary epithelial cell lines (boMECs) are mainly used throughout the scientific community: MAC-T (established in 1991) [44], BME-UV1 (established in 2001) [45], and PS (established in 2015) [46]. It is important to understand that these established boMECs are inherently different from primary cells and from each other, especially the MAC-T and PS *vs.* BME-UV1 cells [47, 48]. In fact, the bovine mammary alveolar and ductal epithelium consists of two layers of epithelial cells, a luminal and a basal layer, where in between so-called mammary ductal macrophages are situated [49]. MAC-T and PS cells originate from the basal mammary gland epithelial layer, whereas BME-UV originate from the luminal layer [48].

### Preclinical mouse models to evaluate endolysin activity

Mouse models have been used as preclinical tools to provide proof-of-concept of new antimicrobial agents. A specific example thereof is the mouse mastitis model, in which mastitis pathogens are inoculated through the teat orifice with a blunted pediatric needle into the mammary ducts (Fig. 3E) [50]. The use of these preclinical models has many advantages in comparison with dairy cows [50, 51]. First of all, mice are cost-effective, easy to house and have a short generation time. Secondly, there are many anatomical similarities between the fourth inguinal mammary gland pair of mice and the udder of cows. Indeed, both species contain functionally and anatomically separated glands that consist of one main milk duct ending on the top of the nipple in one teat orifice. In addition, this inguinal gland pair in mice also contains lymph nodes that similarly occur in cows. Thirdly, there are many analytical tools and immunological reagents available for mice, which is rather limited for cows. Despite these advantages and similarities between the mammary glands of mice and cows, there are obviously also some fundamental differences [50, 51]. The composition of the milk is different and the cow’s udder contains more phagocytic cells under physiological conditions than the mammary gland of mice. Furthermore, mice undergo ‘forced weaning’ 1 h before administration of the intraductal inoculation, which means that the pups are permanently removed. This initiates the involution of the lactating mammary gland, accompanied by a physiological influx of macrophages in the alveolar lumen. Whereas dairy cows are still milked throughout mastitis, this is not done in mice. Mice are small in comparison to cows and their mammary gland tissue is sensitive to an intraductal inoculation, which can result in sepsis caused by the mastitis pathogens [51]. Taken together, the mouse mastitis model is a valid tool to deliver preclinical proof-of-concept of new antimicrobials—including endolysins—that target bovine mastitis pathogens in the lactating bovine mammary gland. This mouse model comes with certain limitations which should be considered when data from mice are translated towards the target species (i.e., the dairy cow).

## Search strategy

This state-of-the-art review conducted an exhaustive exploration through PubMed (http://www.ncbi.nlm.nih.gov/pubmed) and Google Scholar (https://scholar.google.com/) utilizing a targeted search strategy with keywords such as “endolysin”, “bovine mastitis”, and additional relevant terms. The search aimed to identify articles focusing on endolysins characterized or engineered in the context of Gram-positive bovine mastitis. The evaluation process involved a thorough examination of the title and abstract of the obtained hits, with a subsequent retrieval and detailed assessment of articles meeting the specified criteria. Amino acid sequences were sourced either from the NCBI GenBank (https://www.ncbi.nlm.nih.gov/genbank/) or extracted from supplementary materials provided in the selected articles.

## Review

**Fig. 4:**
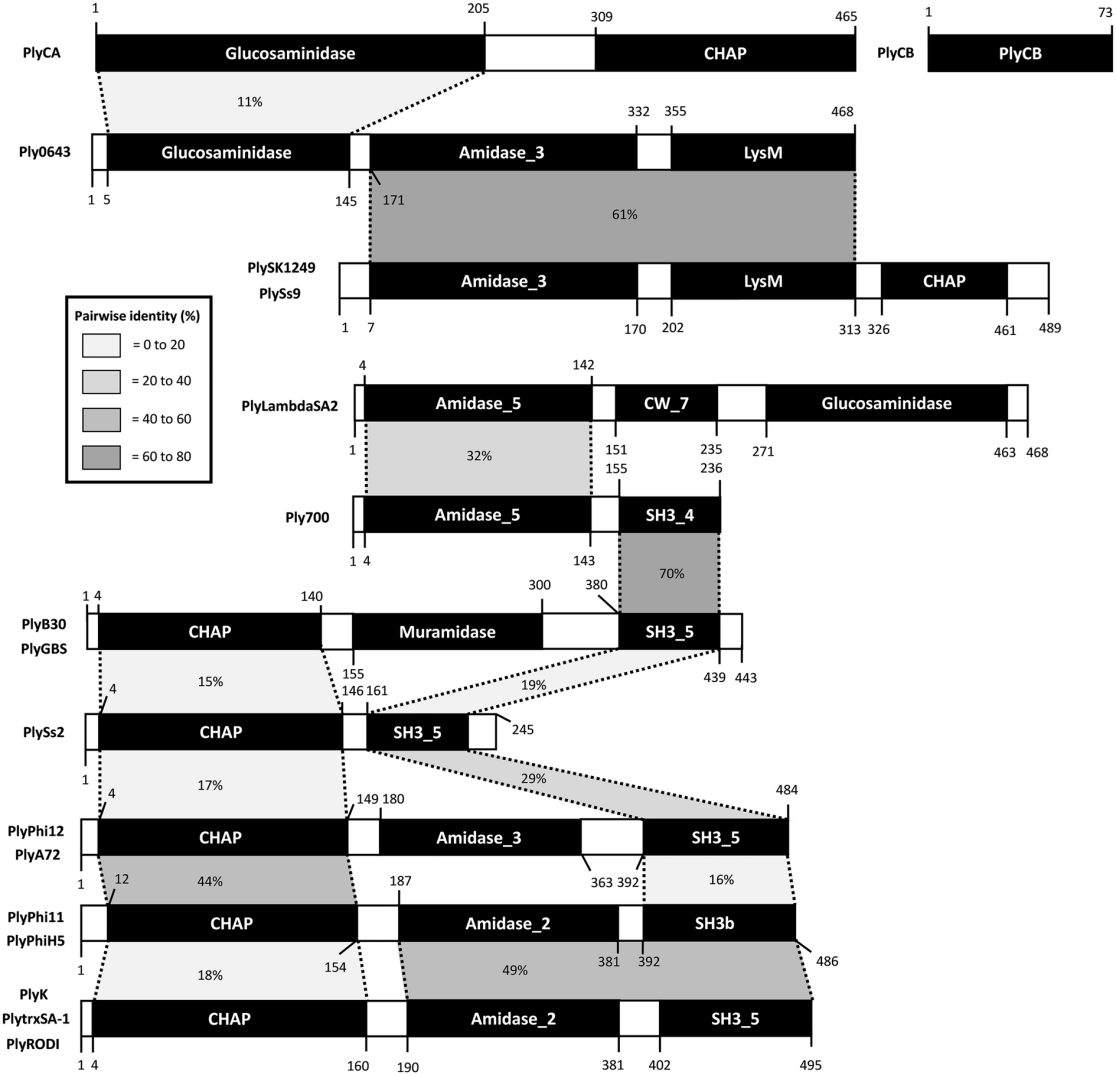
Schematic representation of primary protein structures with annotated enzymatically active domains and cell wall-binding domains. Percentages of pairwise identities between subdomains are presented by means of a grey scale with a 20% interval. Pairwise identities were determined by multiple sequence alignments in Clustal Omaga (MAFFT algorithm) analyses on the protein sequences derived from the NCBI GenBank, or sequences extracted from the supplementary materials provided in the articles that were selected. PlyC is a multimeric endolysin that is composed of the PlyCA and PlyCB subdomains, which are translated from separate genes

To facilitate a rigorous comparative analysis, a multiple sequence alignment was executed using Clustal Omega and the MAFFT algorithm, generating a Pearson/FASTA output [52]. The percentage values presented in this review were consistently derived from the percentage identity matrix of the aligned amino acid sequences. A schematic representation of the primary protein structures has been created to cluster and visualize the homologous wild-type endolysins that have ≥ 95.0% protein sequence similarity, that also shows pairwise homology of their individual subdomains (Fig. 4). Literature will be discussed per homologous group to allow straightforward comparison of the wild-type endolysins, going from the top to the bottom of this schematic representation. Thereafter, the engineered endolysins are discussed in chronological order according to their year of publication. A general overview is provided (Table 1).

To improve uniformization of the endolysin nomenclature that is currently lacking in the field [53] (*e.g.*, LysK, PlyK and phage K endolysin all refer to the same endolysin), a clear distinction was made between the wild-type and engineered endolysins in this work. Wild-type endolysins will always be preceded by Ply (phage endolysin), whereas engineered endolysins will be preceded with Cly (chimeric endolysin). This nomenclature has been proposed and used by most research groups in the USA and Asia [16, 27, 54–57].

**Table 1:** Overview of the endolysins that have been characterized in the context of Gram-positive bovine mastitis

	*S. uberis*					*S. dysgalactiae*				
	Qualitative	TRA (% ΔOD_600_)	TKA (Δlog_10_ CFU/mL)	MIC (µM)	In vivo (Δlog_10_ CFU/g)	Qualitative	TRA (% ΔOD_600_)	TKA (Δlog_10_ CFU/mL)	MIC (µM)	In vivo (Δlog_10_ CFU/g)
**Homologous wild-type endolysins**										
PlyC	ND	ND	ND	ND	ND	ND	40–50 [55]	ND	ND	ND
Ply0643	ND	60–80 [63]	ND	ND	ND	ND	60–80 [63]	ND	ND	ND
PlySK1249 & PlySs9	+ [16]	± 60 [16]	± 2.50 [16]	± 0.50 [16]	ND	ND	15–65 [37]	± 2.00 [37]	ND	ND
PlyLambdaSA2	+ [35]	ND	> 4.00 [35]	ND	± 1.50 [35]	+ [35]	ND	± 3.50 [35]	ND	± 2.20 [35]
Ply700	ND	+ [32]	< 1.00 [32]	ND	ND	ND	20–90 [32]	ND	ND	ND
PlyGBS & PlyB30	+ [35]	ND	± 0.50 [35]	ND	± 2.10 [35]	+ [35]	ND	± 1,50 [35]	ND	± 0.00 [35]
PlySs2 (a.k.a. CF-301)	+ [16]	± 60 [16]	± 1.10 [16]	> 5.00 [16]	ND	ND	ND	± 2.70 [31]	ND	ND
Plyphi12 & PlyA72	ND	ND	ND	ND	ND	ND	ND	ND	ND	ND
PlyPhi11 & PlyPhiH5	ND	ND	ND	ND	ND	ND	ND	ND	ND	ND
PlyRODI & PlytrxSA-1 & PlyK	ND	ND	ND	ND	ND	− [89]	ND	ND	ND	ND
**Engineered endolysins**										
ClyR	ND	10–40 [55]	ND	ND	ND	+ [55]	80–90 [55]	> 4.00 [55]	ND	ND
ClyNC5	+ [31]	80–90 [31]	> 4.00 [31]	ND	> 4.00 [95]	ND	70–80 [31]	± 1.70 [31]	ND	ND
ClyλSA2-PlyK-SH3 & ClyλSA2-Lyso-SH3	+ [20]	10–20 [20]	ND	ND	ND	ND	ND	ND	ND	ND
ClyK-L(-PTD) & ClyL-K(-PTD)	ND	ND	ND	ND	ND	ND	ND	ND	ND	ND
Cly109	ND	ND	ND	ND	ND	ND	ND	ND	ND	ND
ClyCHAPK_CWT-LST & ClyM23LST(L)_SH3b2638	ND	ND	ND	ND	ND	ND	ND	ND	ND	ND

	*S. agalactiae*					*S. aureus*				
	Qualitative	TRA (% ΔOD_600_)	TKA (Δlog_10_ CFU/mL)	MIC (µM)	In vivo (Δlog_10_ CFU/g)	Qualitative	TRA (% ΔOD_600_)	TKA (Δlog_10_ CFU/mL)	MIC (µM)	In vivo (Δlog_10_ CFU/g)
**Homologous wild type endolysins**										
PlyC	ND	< 10 [55]	ND	ND	ND	ND	ND	ND	ND	ND
Ply0643	ND	60–80 [63]	ND	ND	± 3.30 [63]	ND	ND	ND	ND	ND
PlySK1249 & PlySs9	ND	15–65 [37]	± 1.50 [37]	ND	ND	ND	< 10 [37]	ND	ND	ND
PlyLambdaSA2	+ [35]	ND	> 1.50 [35]	ND	± 2.00 [35]	− [28]	ND	ND	ND	ND
Ply700	ND	± 10 [32]	ND	ND	ND	ND	< 10 [32]	ND	ND	ND
PlyGBS & PlyB30	+ [35]	ND	± 2.00 [69]	ND	± 4.50 [35]	ND	< 10 [69]	ND	ND	ND
PlySs2 (a.k.a. CF-301)	ND	20–40 [70]	> 4.00 [70]	9.50 [70]	ND	+ [31]	0–30 [70]	> 4.00 [70]	0.59 [70]	ND
Plyphi12 & PlyA72	ND	ND	ND	ND	ND	ND	ND	ND	1.47 [41]	ND
PlyPhi11 & PlyPhiH5	ND	< 10 [84]	ND	ND	ND	ND	80–90 [36]	> 4.00 [36]	ND	ND
PlyRODI & PlytrxSA-1 & PlyK	− [89]	ND	ND	ND	ND	+ [89]	± 30 [29]	> 4.00 [41]	0.57 [41]	> 3.00 [41]
**Engineered endolysins**										
ClyR	ND	30–40 [55]	< 3.00 [55]	ND	ND	ND	5–30 [55]	ND	ND	ND
ClyNC5	ND	40–50 [31]	± 1.50 [31]	ND	ND	+ [31]	< 10 [31]	< 0.50 [31]	ND	ND
ClyλSA2-PlyK-SH3 & ClyλSA2-Lyso-SH3	ND	10–20 [20]	ND	ND	ND	+ [20]	80–90 [20]	± 1.50 [28]	ND	± 3.30 [28]
ClyK-L(-PTD) & ClyL-K(-PTD)	ND	ND	ND	ND	ND	+ [43]	40–70 [43]	ND	≤ 0.2 [43]	≥ 3.40 [43]
Cly109	ND	ND	ND	ND	ND	+ [40]	ND	> 4.00 [40]	≤ 0.4 [40]	ND
ClyCHAPK_CWT-LST & ClyM23LST(L)_SH3b2638	ND	ND	ND	ND	ND	ND	ND	≤ 3.00 [38]	< 1.0 [38]	ND

A tabular overview of homologous wild-type and engineered endolysins characterized against bovine mastitis-causing pathogens, including *Streptococcus uberis*, *Streptococcus dysgalactiae*, *Streptococcus agalactiae* and *Staphylococcus aureus*, which should be interpreted cautiously due to variations in testing conditions between the different works from which these results were derived, all of which are cited. Qualitative assays involve spot-on-plate, spot-on-lawn, and zymogram assays; quantitative assays include turbidity reduction assay (TRA), time kill assay (TKA), and determination of minimal inhibitory or bactericidal concentration (MIC/MBC). The in vivo condition reflects results obtained in the mouse mastitis model only. ‘ + ’ and ‘−’ denote positive and negative results for qualitative assays, whereas ‘ ± ’ and ‘ND’ signify approximate and unknown results (not determined), respectively

### Wild-type endolysins evaluated against bovine mastitis-causing streptococci

#### PlyC—discovered in 1957 by Richard M. Krause (USA)

PlyC (*a.k.a.* C1 lysin) was discovered in the *Streptococcus dysgalactiae* infecting C1 bacteriophage [58]. It is a multimeric endolysin consisting of the two gene products PlyCA and PlyCB that are linked by salt bridges and/or Van der Waals forces [27, 59]. PlyCA serves as the catalytic domain (containing a GyH and CHAP domain), whereas PlyCB forms an octamer which binds the streptococcal peptidoglycan. PlyC showed promising in vitro activity against *Streptococcus pyogenes* by eliminating 10^6^ CFU in 5 s using only 10 ng enzyme [60]. It was also found effective in mice to protect the oral cavity from colonization by *Streptococcus pyogenes* [60]. Furthermore, PlyC could eradicate streptococci from the oral cavity of mice within 2 h if the oral *mucosae* were infected a few days before with 10^7^ CFU *S. pyogenes*. Besides these observations made for *S. pyogenes*, activity against *Streptococcus uberis* and *S. dysgalactiae* has also been reported [42, 61]. The lysis speed was evaluated by TRA for 250 U (units) enzyme and was in the range of 0.6–1.0 ΔOD_600nm_/min for *S. pyogenes*, whereas this was reduced for *S. dysgalactiae* with an observed ΔOD_600nm_/min of 0.1–0.2. In a follow-up toxicity study, bovine PMN were isolated from blood samples of 12 healthy, mid-lactation, primiparous cows and incubated with increasing concentrations of maximally 50 µg/mL PlyC during up to 2 h. PlyC was non-toxic for bovine blood PMN as evaluated by unchanged lactate dehydrogenase levels [42]. Furthermore, the PMN oxidative burst was also not affected. Both in vitro observations indicate that PlyC may not interfere with the function of PMN during the inflammatory response in the bovine udder. Another remarkable feature of PlyC is the ability to cross the cell barrier of epithelial cells (*i.e.*, shown for human adenocarcinoma A549 cells) due to interactions of PlyCB with phosphatidylserine [62]. PlyC can lyse *S. pyogenes* inside these latter mentioned cells in a dose-dependent manner. Of note, phosphatidylserine is usually only present on the inside of the epithelial cell membrane, but it is believed that these interactions can occur on the outer cell membrane through a cellular recycling mechanism.

#### Ply0643—discovered in 2022 by the Huochon group (China)

Ply0643 was discovered in the *Streptococcus agalactiae* prophage S. *a* 04 [63]. It consists of a N-terminal glucosaminidase, a C-terminal LysM CBD and a middle position amidase_3, the latter two sharing 61% protein sequence similarity with that of PlySK1249 and PlySs9 (Fig. 4). This endolysin was found enzymatically effective in a TRA by reducing the OD_600nm_ of 20 bovine *S. agalactiae* strains, as well as one strain of *S. uberis* and *S. dysgalactiae* with 60 to 80% at 30 µg/mL after 1 h [63]. Moreover, the same concentration of Ply0643 reduced the OD_600nm_ of *S. agalactiae* in 1 h by 80% at pH 6.0 and 7.0, which is consistent with the pH of raw milk. The physiological pH of milk ranges from 6.5 to 6.7 and can be > 7.0 in mastitic cows. Ply0643 was further evaluated in vivo in a murine mouse model for *S. agalactiae* mastitis and showed a Δlog_10_ of 2.4 × 10^2^ CFU/g tissue in the murine mammary glands after 24 h, as well as reduced levels of interleukin (IL)-1β, IL-6 and mouse IL-8 [63]. These results are consistent with a reduced immune cell influx evaluated on hematoxylin & eosin (H&E) stained mammary gland sections. Treatment was given intramammarily, but soon (*i.e.*, 1 h) post-infecting the third and fourth gland pairs with 1.75 × 10^4^ CFU *S. agalactiae*. Glands on the left side received endolysin treatment (*i.e.*, 100 µg/gland), whereas glands on the right side received buffer as placebo treatment. The anatomical relevance of using the third gland pair of mice as a model for mastitis in dairy cows can be questioned, given their axillary instead of inguinal position [50, 51].

#### PlySK1249 and PlySs9—discovered in 2013 and 2020 by the Resch (USA) and Nelson (USA) groups, respectively.

PlySK1249 was discovered in the *S. dysgalactiae* prophage SK1249 and contains an N-terminal amidase_3, a middle LysM and a C-terminal CHAP [37]. It has been shown that the amidase_3 and CHAP domains synergize for peptidoglycan digestion and bacteriolysis [64]. PlySK1249 was lytic for one *S. dysgalactiae* and four *S. agalactiae* isolates in a TRA, as OD_600nm_ reductions were obtained in the range of 15 to 65% with 3.3 U/mL within 15 min. Interestingly, *S. uberis* and *Streptococcus suis* were also susceptible under the same conditions, with 20% and 30% decreases in OD_600nm_, respectively. Two *Staphylococcus aureus* strains were additionally challenged, albeit found non-susceptible (*i.e.*, ΔOD_600nm_ of < 10%). The optimal pH was found to be between pH 7 and 8.5, with a 50% decrease in OD_600nm_ achieved at these pH values in 15 min with 3.3 U/mL and *S. dysgalactiae* SK1249 as the target. The bactericidal activity was further evaluated in a kinetic TKA with 3.3 U/mL against one isolate of *S. agalactiae* and *S. dysgalactiae*, which caused 1.5 and 2.0 log_10_ reductions within 15 min, respectively. Subsequently, the activity of PlySK1249 was further investigated in a mouse model of *S. agalactiae* bacteremia. The animals were challenged intraperitoneally with 10^6^ CFU of *S. agalactiae* and 20% survived within 72 h, whereas this was increased by 60% if the infected mice received daily injections of 45 mg/kg PlySK1249. Another endolysin, named PlySs9, shares 96.5% protein sequence similarity with PlySK1249 and was discovered in a prophage element from a *S. suis* isolate belonging to serotype 9 (Fig. 4) [16]. The activity of the wild-type endolysin, as well as of the individual domains, was evaluated against *S. uberis* and *S. suis* in comparison with a second *S. suis*-derived endolysin PlySs2. PlySs9 was found more potent than PlySs2 against reference isolate *S. uberis* 0140J, as 0.5 µM PlySs9 in comparison with an equimolar amount of PlySs2 showed: (i) a slightly higher ΔOD_600nm_ by TRA (*i.e.*, 0.05 after 1 h), one additional Δlog_10_ by TKA (*i.e.*, 2.54 ± 0.08 *vs*. 1.09 ± 0.37 after 2 h), and (iii) a lower MIC (*i.e.*, 0.48 ± 0.16 *vs.* > 5 µM). Five clinical and subclinical bovine mastitis-derived *S. uberis* strains were similarly challenged by TRA, which showed that all strains were susceptible to both endolysins, but PlySs9 could again decrease the OD_600nm_ more. Molecular dissection of these endolysins in EAD and CBD subdomains resulted in nearly complete loss of killing and binding activity, respectively.

#### PlyλSA2—discovered in 2007 by the Baker group (USA)

PlyλSA2 has been discovered in the *S. agalactiae* prophage LambdaSA2 [65]. PlyλSA2 consists of an N-terminal amidase_5, a middle CW_7 and a C-terminal glucosaminidase. The activity of PlyλSA2 has been comparatively characterized with the non-homologous PlyB30 (Fig. 4) [35]. Both endolysins were qualitatively evaluated by spot-on-lawn, in which 0.12 ± 0.01 and 0.26 ± 0.01 µg caused a halo against *S. dysgalactiae* and *S. uberis*, respectively. An approximate tenfold dose was needed for PlyB30 to observe a similar halo against these strains, which made the authors conclude that PlyλSA2 was the most potent endolysin. *S. agalactiae* was challenged in similar fashion with both endolysins but was found less susceptible, given that 1.72 ± 0.58 and 4.69 ± 1.12 µg were needed to observe halos. The optimal pH was found to be consistent with that of mastitic and healthy raw cow’s milk, *i.e.* in the range of 7.0 to 7.5 and around 6.5 for PlyλSA2 and PlyB30, respectively. Increasing concentrations of Ca^2+^ (*i.e.*, 0.1, 1.0 & 10.0 mM) inhibited the activity of PlyλSA2 as was evaluated by TRA against *S. dysgalactiae*, whereas that of PlyB30 was enhanced. More specifically, a ± 50% reduction in residual activity was observed at 10 mM Ca^2+^ for 25 µg/mL PlyλSA2. In contrast, this was a ± 50% increase at 1 mM Ca^2+^ for 100 µg/mL PlyB30. Of note, the most abundant divalent cation in cow milk is calcium with a concentration of approximately 3.0 mM unbound or ‘free’ Ca^2+^. A kinetic TKA was performed in UHT-milk during 3 h and 100 µg/mL of both endolysins, which caused log_10_ reductions of all three streptococcal species at the end of the assay, although the reduced activity of PlyB30 *vs.* PlyλSA2 was again observed. CFU levels were increased by 1.5 to 2.0 log_10_ after 3 h in these UHT-milk conditions in the wells that received no treatment (the negative control). At an inoculum dose of 10^3^ CFU/mL, PlyλSA2 and PlyB30 caused approximately 3.5 and 2.5 Δlog_10_ at a concentration of 100 µg/mL of *S. dysgalactiae*, respectively. This was > 4.0 and > 1.5 for PlyλSA2, and, approximately 2.0 and 0.5 for PlyB30 against *S. uberis* and *S. agalactiae*, respectively. Thus, the reduced activity that was observed against *S. agalactiae* in the spot-on-lawn assay was likewise visible in the kinetic TKA. Given that both endolysins attack different bonds in the streptococcal peptidoglycan, their potential synergy was further investigated. PlyλSA2 and PlyB30 showed synergy (*i.e.*, Σ 0.42 ± 0.09) against *S. dysgalactiae* in an in vitro checkerboard-based assay. Σ represents the fractional inhibitory concentration index value, which indicates synergy if Σ < 0.50. Synergy of both endolysins against the *S. uberis* and *S. agalactiae* strains was not determined. Both endolysins were subsequently tested individually and as a potentially synergistic combination in a mouse mastitis model side-by-side against all three streptococcal pathogens. The doses used per gland consisted of 25 and 250 µg for either PlyλSA2 or PlyB30, respectively, whereas the synergistic combination consisted of 12.5 and 125 µg PlyλSA2 and PlyB30. It should be noted that intramammary therapy was given as soon as 45 min after intraductally inoculating only 10^2^ CFU pathogenic bacteria in the murine mammary glands. For all three streptococcal species, bacterial concentrations in the mammary glands were significantly reduced 24 h after bacterial challenge in the range of 1.5 to 4.5 Δlog_10_, except for PlyB30 against *S. dysgalactiae* that showed no log_10_ reduction. Indeed, the latter contrasted the in vitro results of PlyB30 for *S. dysgalactiae*, which thus could not be retrieved in vivo. Furthermore, PlyB30 unexpectedly caused higher log_10_ reductions for *S. agalactiae* than for *S. uberis*, of approximately 4.5 *vs.* 2.1 Δlog_10_, respectively. The most surprising finding, however, was that the synergistic action of both lysins was lost in the murine mammary gland.

#### Ply700—discovered in 2008 by the Kerr group (USA)

Ply700 was discovered after inducing a prophage in the genome of *S. uberis* ATCC 700407 with mitomycin C [32]. It consists of an N-terminal amidase_5 and C-terminal SH3_4, of which the latter has approximately 70% protein sequence similarity with the CBD of PlyGBS and PlyB30 (Fig. 4). This wild-type endolysin has an optimal activity against *S. uberis* at a pH of 6.0 to 7.0, which is consistent with that of raw cow’s milk. At these pH values, a 40% decrease in turbidity was observed by TRA with 20 µg/mL enzyme in 25 min. In addition, it was also described that a 10 mM Ca^2+^ concentration is required to exploit its maximal activity. As observed by TRA using the same endolysin concentration, Ply700 caused a ± 60% decrease in turbidity of *S. uberis* in the presence of 10 mM Ca^2+^, whereas this was only ± 25% at a tenfold lower Ca^2+^ concentration. Ply700 was found effective by TRA against one *S. dysgalactiae* and ten *S. uberis* strains, in comparison with one *S. agalactiae* strain that only showed minor activity. More specifically, the ΔOD_600nm_ was in the range of 20 to 90% for *S. uberis* and *S. dysgalactiae*, whereas this was only ± 10% for the *S. agalactiae* isolate, all evaluated with 50 µg/mL enzyme during 30 min. The authors also determined the activity of Ply700 against *S. uberis* in UHT whole milk with a non-kinetic TKA. They found that spiking the milk with 50 µg/mL Ply700 could kill 30% of a *S. uberis* strain within 15 min, if 4.5 × 10^3^ CFU/mL were added. Additionally, the C-terminal SH3_4 domain was fused to GFP and binding to *S. uberis* was investigated in the presence and absence of Ca^2+^. This revealed that binding of the SH3_4 domain was calcium-dependent, as addition of 10.0 mM Ca^2+^ increased the binding by approximately 4 × in comparison to the condition where Ca^2+^ was not supplemented. The latter phenomenon is similarly reported for PlyB30 [66].

#### PlyB30 and PlyGBS—discovered in 2004 and 2005 by the Baker (USA) and Fischetti (USA) groups, respectively

PlyB30 has been derived from the virulent *S. agalactiae* phage B30 [66]. It consists of an N-terminal CHAP, a central muramidase and a C-terminal SH3_5 domain, of which the former has been characterized as the major contributor of the lytic activity observed with *S. agalactiae* [67]. The activity of PlyB30 has been comparatively characterized with the non-homologous PlyλSA2, as already discussed. A homologous endolysin, PlyGBS, has likewise been discovered in the virulent *S. agalactiae* phage NCTC 11261 [68]. PlyGBS dropped the OD_600nm_ in a TRA to the baseline within 10 min at 40 U of a *S. agalactiae* isolate. This rapid rate of cell lysis led to a ± 2.0 log_10_ decrease of the same *S. agalactiae* isolate, observed in a subsequent TKA. Furthermore, the lytic spectrum of PlyGBS was evaluated at 15 U during 60 min by TRA against six *S. agalactiae* isolates, as well as one isolate of S. *dysgalactiae* and *S. aureus*. Like the first observation, PlyGBS lysed all tested *S. agalactiae* serotypes and the *S. dysgalactiae* isolate in vitro, but not *S. aureus*. To further evaluate the potency of PlyGBS in vivo, mice were challenged vaginally with 10^6^ CFU *S. agalactiae* [68]. After 24 h, mice received vaginal treatment with either endolysin buffer or 10 U PlyGBS and were swabbed 2 and 4 h post-treatment. The treated animals showed a Δlog_10_ of 3.0 at both the 2 and 4 h time intervals compared to the buffer control. In addition, mice were also challenged orally and nasally with 10^8^ CFU *S. agalactiae* to determine if PlyGBS can be used to reduce colonization of the upper respiratory tract. Mice treated with 10 U PlyGBS exhibited a reduction in *S. agalactiae* colonization of approximately 0.5 and 1.5 Δlog_10_ at both the 2 and 24 h swabbing intervals, in comparison with the buffer control. In a follow-up study, DNA mutagenesis methods were explored to produce hyperactive PlyGBS mutants with 18- to 28-fold increased lytic activity against *S. agalactiae*, evaluated by TRA [69]. Using the same mouse vaginal model, one of these hyperactive mutants (*i.e.*, PlyGBS90-1) reduced *S. agalactiae* with ± 4.0 Δlog_10_ at 4 h post-treatment using a single dose of only 30 nmol.

### Wild-type endolysins evaluated against bovine mastitis-causing staphylococci

#### PlySs2—discovered in 2013 by the Fischetti group (USA)

PlySs2, a.k.a. CF-301 or Exebacase, is by far the best characterized Gram-positive endolysin with in vivo confirmed broad lytic activity against a large variety of both streptococci and staphylococci [16, 70–79]. It was considered a lead therapeutic candidate in the endolysin field, evaluated in clinical trials by ContraFect. This company aimed a therapeutic application in humans suffering from sepsis and right-heart endocarditis caused by methicillin-resistant *S. aureus* (MRSA), as well as chronic prosthetic joint infections of the knee [73]. However, the company recently filed for bankruptcy. Nevertheless, PlySs2 reached breakthrough therapy in phase III clinical trials for the former application that were discontinued due to a lack of statistical power [74]. ContraFect stated this was possibly due to an imbalance in the baseline disease severity of patients in the PlySs2 *vs.* placebo group, with more patients having an extremely poor prognosis (*i.e.*, APACHE II score above 15) and increased risk of mortality in the PlySs2 treated group. Of note, PlySs2 was only administered to patients as an add-on to the currently used standard-of-care antibiotics [74].

PlySs2 was discovered in a prophage element of a *S. suis* isolate belonging to serotype 2 [16, 75]. Multiple studies report that relatively low concentrations of PlySs2 can efficiently lyse *S. aureus*, *S. agalactiae, S. dysgalactiae* and *S. uberis* below detection limits in different kinds of qualitative and quantitative assays, including TRAs, TKAs, MIC/MBCs and in vivo studies [16, 70–73]. This endolysin consists of an N-terminal CHAP and a C-terminal SH3_5. Two studies performed a molecular dissection of PlySs2 to study the EAD and CBD separately [15, 16]. The EAD alone lost its catalytic activity completely, as was observed by combining TRAs and TKAs. This indicates the necessity for the SH3_5 domain to be present to exploit the full functionality of PlySs2. On the other hand, the SH3_5 domain was fused to either GFP or conjugated with Alexa Fluor 555 to study binding to several streptococcal and staphylococcal isolates. It was qualitatively shown by fluorescence microscopy that the SH3_5 domain can bind *S. agalactiae*, *S. dysgalactiae* and *S. suis*, but remarkably not *S. uberis* and *S. aureus*. The authors state that this is not a surprising finding, but rather underscores the importance of the EAD to be present for full binding functionality of PlySs2 [16]. For completion, PlySs2 SH3_5 has also been attributed several eukaryotic cell penetrating properties [76]. To proceed, PlySs2 has been characterized with potent biofilm eradicating activity [77, 78]. Minimum biofilm-eradicating concentration (MBEC) assays on 95 *S. aureus* strains revealed a 90% MBEC (MBEC_90_) value of ≤ 0.25 μg/mL. This was evaluated on biofilms formed on polystyrene, glass, surgical mesh, catheters and in human synovial fluid. Of note, one *Streptococcus agalactiae* biofilm was also found sensitive to disruption, with an MBEC_90_ value of 0.50 μg/mL. PlySs2 also exhibited substantially increased potency (32 to ≥ 100-fold) in human blood *vs.* laboratory testing media in three complementary microbiological testing formats (*i.e.*, MICs, TKAs and checkerboard synergy) [79]. More specifically, PlySs2 acts synergistically with two key human blood factors: serum lysozyme and albumin. For lysozyme, it is reasoned that the synergistic effect is obtained through initial disruption of the staphylococcal peptidoglycan which makes it sensitive to the subsequent action of lysozyme, whereas the synergistic action of albumin occurs through high affinity. Indeed, both *S. aureus* and PlySs2 have high affinity for albumin, causing them to colocalize in blood. The latter might be interesting from a bovine mastitis point of view, as raw milk contains lactalbumin which might exert a similar synergistic effect.

#### PlyPhi12 and PlyA72—described in 2004 and 2021 by the Bierbaum (Germany) and Garcia (Spain) groups, respectively

PlyA72 was discovered in the virulent *S. aureus* phiIPLA35 phage and shares approximately 98.0% protein sequence similarity to PlyPhi12 (Fig. 4) [80]. Both endolysins consist of an N-terminal CHAP, a middle amidase_3 and a C-terminal SH3_5. Although PlyPhi12 was bio-informatically discovered 17 years before PlyA72, it was not further characterized because the endolysin auto-aggregated in the assays performed [80]. Thus, antibacterial assays were only performed later for the homologous endolysin PlyA72 in comparison with PlyRODI [41]. PlyA72 had inferior staphylococcal activity compared to the latter endolysin. More specifically, LysRODI displayed lower MIC values in comparison with PlyA72 of 0.57 *vs.* 1.47 µM, respectively, against two bovine mastitis-derived *S. aureus* strains. The biofilm eradicating potential of PlyA72 was also reduced if compared to PlyRODI, as 7 µM of these endolysins reduced 17% and 94% of a *S. aureus* biofilm in 5 and 1 h, respectively.

#### PlyPhi11 and PlyPhiH5—discovered in 2006 and 2008 by the Donovan (USA) and Garcia (Spain) groups, respectively

PlyPhi11 was discovered in the virulent phage phiIPLA88 infecting *S. aureus* [36]. It consists of an N-terminal CHAP, a middle amidase_2 and C-terminal SH3_5. It was shown that the middle amidase_2 has minor contributions to PlyPhi11’s activity, as its deletion did not result in loss of activity against *S. aureus* in a TRA [81]. The same has been reported for PlyK, which has an equal molecular architecture [82]. In fact, it has been shown that the amidase_2 or _3 in staphylococcal wild-type endolysins has an auxiliary role and mainly possesses a binding function [83]. A PlyPhi11 dose of 25.0 µg was able to lyse mastitis-causing staphylococci in a 1 h pending TRA, such as one *S. aureus*, one *S. chromogenes* and one *Staphylococcus simulans* isolate [84]. The activity was similarly tested against one mastitis-causing *S. agalactiae* isolate, but no activity was observed. The authors therefore claimed PlyPhi11 to be a staphylococcal endolysin. The activity of PlyPhi11 was evaluated by TRA under the same conditions at the pH (*i.e.*, 6.7) and Ca^2+^ concentration (*i.e.*, 3 mM) of milk, at which peak activity of PlyPhi11 was observed. No subsequent assays in UHT-milk or raw milk were conducted. However, another lysin named PlyPhiH5 that shares > 97.0% protein sequence similarity to PlyPhi11 (Fig. 4) has been reported to reduce *S. aureus* in UHT whole milk from 10^6^ CFU/mL to undetectable levels after 4 h starting at a concentration of 1.6 µM [36]. Similarly to PlyPhi11, the lytic activity of PlyPhiH5 was found optimal around the pH of milk and no activity was observed against streptococci, all evaluated by TRA using 15 U/mL enzyme. Interestingly, the authors reported increased activity of PlyPhi11 on bovine mastitis *S. aureus* isolates in comparison with those from human origin, more specifically a specific activity of 11.3 ± 1.7 *vs.* 7.5 ± 2.9, respectively.

#### PlyK, PlytrxSA-1 and PlyRODI—discovered in 2005, 2016 and 2021 by the Ross (Ireland), Jingyun (China) and Garcia (Spain) groups, respectively

PlyK, PlytrxSA-1 and PlyRODI were discovered in the virulent *S. aureus* phages K [85–88], IME-SA1 [89] and phiIPLA-RODI [90, 91], respectively. These endolysins consist of an N-terminal CHAP, a middle amidase_2 and a C-terminal SH3_5. PlyK is one of the earliest discovered endolysins with confirmed qualitative activity against three bovine mastitis *S. aureus* in a spot-on-plate assay [29]. PlytrxSA-1 has likewise been evaluated in a spot-on-lawn assay at 6 µg/µL against 100 *S. aureus* isolates, of which 43 showed a halo. This included bovine mastitis-derived strains. Also, one isolate of bovine mastitis *S. agalactiae* and *S. dysgalactiae* were similarly challenged, but no activity was observed [89]. Interestingly, PlytrxSA-1 has been used to treat three bovine udder quarters infected with *S. aureus* (*i.e.*, milk with a positive culture for *S. aureus* and somatic cell count (SCC) > 500,000 cells/mL). Each udder quarter received a daily inoculation of 20.0 mg PlytrxSA-1 during three consecutive days. By day 3, *S. aureus* CFU counts as well as milk SCC were reduced to the limit of detection and milk consistency appeared back to normal. Unfortunately, this study did not include an appropriate negative control (*i.e.*, untreated *S. aureus* infected udder quarters). The latter experimental design makes it hard to state that the observed clinical improvement is (at least partially) due to the PlytrxSA-1 treatment. PlyRODI has been discovered and comparatively studied with PlyA72 [41]. Both endolysins showed optimal activity in a TRA against a bovine *S. aureus* isolate at the pH and calcium concentration of milk and their lytic activity increased by a twofold at higher calcium concentrations, in line with PlyK for PlyRODI [92]. Both endolysins were further compared in a MIC assay against two bovine mastitis-derived *S. aureus* isolates, in which PlyRODI yielded a lower MIC value (0.57 µM) than PlyA72 (1.47 µM). The results corroborated a subsequent 1 h pending kinetic TKA, in which both enzymes were challenged at 0.1 µM against a bovine mastitis-derived *S. aureus* isolate. PlyRODI eradicated 10^7^ CFU/mL within 1 h, in contrast to PlyA72 which killed 10^3^ CFU/mL. Moreover, PlyRODI could remove 94% of an *S. aureus* biofilm within 1 h, compared to 17% after 5 h for PlyA72. Therefore, only PlyRODI was selected for further in vivo preclinical characterization (*i.e.*, in a zebrafish and mouse mastitis model). In the zebrafish model, PlyRODI was found to be non-toxic and 1.0 µM increased the survival rate with 44.4% at 72 h after intraperitoneal infection with 10^5^ CFU *S. aureus*. In the mouse model for bovine mastitis, murine mammary glands were pre-treated with 24 µg PlyRODI and then challenged with 10^4^ CFU *S. aureus*. A short time interval of 1.5 h was used between the preventive treatment and the *S. aureus* challenge. Mice were euthanatized at 18 h post-*S. aureus* challenge to harvest the mammary glands. This pre-treatment prevented the occurrence of mastitis lesions and reduced the outgrowth of *S. aureus* by 10^3–4^ Δlog_10_. These data show the potential of PlyRODI as a preventive treatment for *S. aureus* mastitis.

### Engineered endolysins evaluated against bovine mastitis-causing streptococci

#### ClyR—engineered in 2015 by the Nelson (USA) and Hongping Wei (China) groups

ClyR was selected from a library consisting of 21 engineered endolysins (*i.e.*, constructed out of seven EADs and three CBDs) against a bovine mastitis *S. dysgalactiae* isolate [55]. This engineered endolysin combines the CHAP of PlyC with the SH3_5 of PlySs2. It caused a ΔOD_600nm_ of ± 0.8 after 30 min against one *S. dysgalactiae* isolate at 25 µg/mL in a TRA, which corresponded to a 4.0 log_10_ reduction in a TKA. Moreover, the same concentration of ClyR also showed activity in a TRA against seven *S. uberis*, six *S. agalactiae* and two additional *S. dysgalactiae* isolates with a ΔOD_600nm_ in the range of 0.1–0.4, 0.3–0.6 and around 0.5, respectively, after 20 min using a dose of 25 µg/mL. Moreover, this activity was found to be calcium independent as there was no reduction observed with the addition of 50 mM ethylenediaminetetraacetic acid (EDTA). ClyR’s activity was also evaluated against *S. agalactiae* and *S. dysgalactiae* in pasteurized raw cow’s milk from both healthy and mastitic cows at 40 µg/mL, causing Δlog_10_ between 1.0 and 3.0 after 1 h. Interestingly, this activity was found to show a 2.0-log_10_ fold increase in the mastitic *vs.* healthy pasteurized milk. The latter is most likely caused by a pH-related effect, as ClyR displayed its highest lytic activity around pH 8.0. When compared to the wild-type endolysin PlyC in a TRA, the activity of ClyR was found to be slightly increased against *S. dysgalactiae* (*i.e.*, additional ΔOD_600nm_ of ± 0.05), but greatly increased against one *S. agalactiae* isolate (*i.e.*, additional ΔOD_600nm_ of ± 0.3). In addition, a broader lytic spectrum was present because several staphylococcal species such as *S. aureus* could be lysed, in contrast to PlyC (*i.e.*, additional ΔOD_600nm_ in the range of ± 0.15–0.35 for 3 *S. aureus* isolates). It has also been shown that ClyR is able to tackle streptococci intracellularly, due to certain cell penetrating properties of PlySs2 SH3_5 [76]. The intracellular uptake of ClyR is believed to happen through caveolin-dependent endocytosis and was shown by confocal laser microscopy, after exposing human adenocarcinoma A549 cells with 100 µg/mL Alexa Fluor 488-labeled ClyR during 40 min.

#### ClyNC5—engineered in 2023 by the Briers, Lavigne and Meyer (all Belgium) groups

The engineered endolysin ClyNC5 was selected from an extensive library comprising over 80,000 theoretical endolysin variants [31]. This library was crafted through the high-throughput DNA assembly platform known as VersaTile [93, 94]. The library underwent a systematic screening process by employing a halo-based assay, wherein bacteriolytic activity was serially assessed through overexpression in individual *E. coli* colonies on agars embedded with either the reference isolate *S. aureus* N305 or *S. uberis* 0140 J. This innovative approach not only facilitated the identification of design rules, but also involved statistical analyses for the robust validation thereof. It was found that CPPs are ideally fused to the N-terminal site, and the engineered construct preferably has a pI in the range 9.05 to 9.65. The standout candidate arising from this screening process was named ClyNC5 and consisted of (from N- to C-terminus) the trans-activator of transcription (TAT) peptide of human immunodeficiency virus (HIV)-1 as CPP, the PlySs2 CHAP, a repeated CW_7 as CBD, and the PlySs9 amidase. ClyNC5 was further evaluated against Gram-positive isolates sourced from (sub)clinically affected cows and had an impressive killing efficacy of 4.05 ± 0.07 Δlog_10_ against *S. uberis* at 0.3 µM and demonstrated comparable activity of 1.50 ± 0.02 and 1.77 ± 0.43 Δlog_10_ against *S. agalactiae* and *S. dysgalactiae*, respectively. In addition, ClyNC5 effectively eradicated approximately 70% of a *S. uberis* biofilm at 1.5 µM, corresponding to 1.17 ± 0.39 Δlog_10_, and displayed intracellular activity at 2.5 µM within MAC-T and PS of 1.62 ± 0.05 and 2.12 ± 0.08 Δlog_10_, respectively. The intracellular presence of ClyNC5 was verified in MAC-T by confocal microscopy. Furthermore, the lead candidate potentiated cloxacillin in raw cow’s mastitic milk at 0.5 µM, which is a beta-lactam penicillin commonly employed for the intramammary treatment of Gram-positive bovine mastitis.

To validate ClyNC5's therapeutic potential as a supplemental therapy to cloxacillin, a preclinical study was conducted using a mouse model for *S. uberis* mastitis [95]. This study involved intramammary infection with a bovine *S. uberis* field isolate of the pathotype GCC ST-5, followed by treatment with cloxacillin combined with varying doses of ClyNC5 (i.e., 23.5 and 235.0 µg) that was administered 12 h post-infection. The hallmarks of mastitis were evaluated mid-treatment, being 16 h post-infection. The results unveiled distinct responder profiles, categorizing mice as either fast (n = 17) or slow (n = 10) responders. In the fast responders, the high-dose combination therapy (i.e., 235.0 µg ClyNC5 + 30.0 µg cloxacillin): (i) reduced the bacterial load by 13,000-fold, (ii) mitigated the intramammary neutrophil influx, and (iii) reduced the pro-inflammatory chemokine IL-8 13-fold. Furthermore, the intramammary immune profile was further complemented by the evaluation of other pro-inflammatory cytokines, chemokines, growth factors and metabolites (*i.e.*, TNF-α, MCP-1, M- & G-CSF as well as IL-1α, -1β, & -6). This similarly revealed an overall dose-dependent reduction of the evaluated markers caused by the supplementation of endolysin ClyNC5 to cloxacillin. Together, both studies provide compelling evidence of ClyNC5’s efficacy as an adjunct to intramammary cloxacillin treatment.

### Engineered endolysins evaluated against bovine mastitis-causing staphylococci

#### ClylSA2-PlyK-SH3 & ClylSA2-Lyso-SH3—Engineered in 2012 by the Donovan group (USA)

ClylSA2-LysK-SH3 and ClylSA2-Lyso-SH3 are two rationally created engineered endolysins that combine the N-terminal streptococcal amidase_5 domain of PlyλSA2 with the C-terminal staphylococcal SH3 domains of either the wild-type endolysin PlyK or lysostaphin, respectively [28]. Of note, lysostaphin is not an endolysin but a bacteriocin. Both engineered endolysins showed qualitative lytic activity in a spot-on-lawn assay against 16 bovine mastitis *S. aureus* isolates, including the bovine mastitis reference strain *S. aureus* N305. The activity of both engineered endolysins has also been evaluated comparatively between *S. aureus* N305 and one isolate of *S. uberis* and *S. agalactiae*, albeit only biochemically in a spot-on-plate assay and via TRA [20]. A concentration of 100 µg/mL had lytic activity against all strains, although the relative specific activity was at least fivefold reduced against both streptococcal isolates. A kinetic TKA was also performed in UHT-milk and 100 µg/mL of both engineered endolysins showed a reduction of the 10^6^ CFU/mL *S. aureus* spiked UHT-milk with a Δlog_10_ of 1.0 to 1.5 after only 1 h. This Δlog_10_ continued to increase for ClylSA2-Lyso-SH3 till 3 h, resulting in a 3.0 Δlog_10_ [28]. Compensatory growth was observed after 1 h for ClylSA2-LysK-SH3, which caused log_10_ values to increase to 6.5 resulting in a difference of only 1.0 Δlog_10_ compared to the negative control. Subsequently, both engineered endolysins were tested in vivo in a murine model of bovine *S. aureus* mastitis using the N305 strain and compared with the natural bacteriocin lysostaphin as a positive control. Treatment with 25 µg/gland was given either only 30 min or 6 h after the inoculation of 10^4^ and 10^2^ CFU *S. aureus*, respectively. The outcome was, however, found to be independent of these variables. More specifically, ClylSA2-LysK-SH3 and ClylSA2-Lyso-SH3 caused a Δlog_10_ of 0.63 and 0.81 at 24 h after the in vivo challenge, respectively. Both engineered endolysins had poor lytic activity in comparison with lysostaphin, which caused a 2.82 Δlog_10_ at 24 h. Given the promising latter result, the authors determined in vitro synergy of lysostaphin in a checkerboard-based assay with both engineered endolysins and found it to be strong (*i.e.*, Σ of 0.46 ± 0.07 and 0.42 ± 0.07 for lysostaphin with ClylSA2-LysK-SH3 and ClylSA2-Lyso-SH3, respectively). Furthermore, the lysostaphin/ClylSA2-LysK-SH3 combination caused a Δlog_10_ of 3.36 in the murine *S. aureus* mastitis model, which was increased compared to lysostaphin and ClylSA2-LysK-SH3 separately (*i.e.*, Δlog_10_ of 2.14 and 0.86, respectively). It should be noted that both experiments cannot directly be compared, as the authors changed some of the parameters of the latter in vivo experiment. Indeed, the inoculation dose (10^3^ CFU), the treatment dose (1:2 diluted) and the endpoint of the experiment (18 h) were altered. Tumor necrosis factor (TNF)-α concentrations were also determined on mammary gland lysates, which followed a decreasing trend correlating with the number of CFU/mL.

#### Triple acting enzymes ClyK-L(-PTD) & ClyL-K(-PTD)—engineered in 2015 by the Donovan group (USA)

ClyK-L was engineered by altering the M23 EAD of the bacteriocin lysostaphin by the CHAP and amidase_2 EADs of the wild-type endolysin PlyK, whereas in the ClyL-K variant these latter mentioned EADs were integrated in between the M23 and SH3b domains of lysostaphin [43]. Both rationally engineered endolysins showed similar biochemical activity in a TRA against one *S. aureus* isolate, but this was increased with ± 0.2 ΔOD_600nm_ if compared to lysostaphin and PlyK. However, an increased MIC-value was sometimes observed for both variants in comparison with one or both parental enzymes, illustrating again the low or inexistent correlation between both assays. For example, lysostaphin yielded a MIC of 0.77 µg/mL against *S. aureus* Newman, whereas this was ≥ 7.0 µg/mL for both engineered endolysins. In addition, ClyK-L was also tested against a biofilm-forming MRSA strain at 100 µg/mL, which reduced the intra-biofilm viability with 24%. ClyK-L and ClyL-K were then further engineered by adding several C-terminal protein transduction domains (PTDs), *a.k.a.* cell penetrating peptides (CPPs), resulting in ClyK-L-PTD & ClyL-K-PTD variants. Subsequently, their intracellular eradicating capacity was evaluated in MAC-T against *S. aureus* ISP479C at 25.0 µg/mL during 2.5 h. Surprisingly, this revealed that addition of a PTD decreased the intracellular killing capacity of the ClyL-K-PTD variant, whereas that of ClyK-L-PTD was not improved. However, the addition of a PTD could reduce the intra-biofilm viability by an additional 16%. One of the ClyK-L-PTD variants was shown to colocalize with *S. aureus* intracellularly as was observed by confocal microscopy. One ClyK-L-PTD variant was selected for in vivo validation in a mouse model for bovine *S. aureus* mastitis, in which mice were intramammarily infected with 100 CFU *S. aureus* and side-by-side treated 30 min thereafter with 25.0 µM of either lysostaphin, ClyK-L or the ClyK-L-PTD variant. Up to six glands per animal were used and animals were euthanized 18 h post-treatment to harvest the mammary glands. The authors found that the ClyK-L-PTD variant, which previously showed intra-biofilm killing activity, caused an additional killing of 2.7 log_10_ in comparison with ClyK-L. Of note, this latter mentioned non-PTD-containing variant only caused a Δlog_10_ of 0.7 in comparison with the phosphate buffered saline (PBS) negative control. The ClyK-L-PTD variant did not perform better than lysostaphin and even had a reduced killing of 0.5 log_10_ in comparison with the latter. TNF-α levels were also determined in mammary gland lysates and followed a correlating trend with the CFUs at 18 h post-treatment.

#### Cly109—engineered in 2020 by the Sangryeol group (South Korea)

Cly109 was hit-to-lead selected from 480 theoretical combinations after high-throughput assembly of various *S. aureus* EADs and CBDs, which were subsequently screened for lytic activity against *S. aureus*, including one bovine mastitis-derived isolate [40]. Cly109 combines the N-terminal CHAP of PlySA12 with the middle amidase_3 and C-terminal CBD of PlySA97 [83, 96]. PlySA12 bears > 96.0% protein sequence similarity to the previously described wild-type endolysins PlyPhi11 and PlyPhiH5. This engineered endolysin was found to be superior to both parental endolysins against *S. aureus* if tested at an equimolar concentration of 300 nM, showing a: (i) > twofold increase in activity evaluated by TRA, (ii) > twofold lower MIC value of 0.37 nM, and (iii) > threefold efficacy in eradicating *S. aureus* biofilm. It had an optimal activity at the pH of milk and a concentration of 0.9 µM eliminated 10^5^ CFU/mL from UHT-milk in only 45 min. The authors reasoned that altering the wild-type CBD from PlySA12 with the amidase_3 + CBD from PlySA97 through their domain swapping method could increase the binding capabilities of the PlySA12 CHAP. Indeed, it has been proposed for amidases in staphylococcal endolysins that these mainly constitute a binding function [83].

#### ClyCHAPK_CWT-LST & ClyM23LST(L)_SH3b2638—engineered in 2018 and 2022 by the Loessner group (Switzerland), respectively

ClyCHAPK_CWT-LST was discovered by high-throughput assembling approximately 170 endolysins from staphylococcal EADs and CBDs, followed by hit-to-lead selection of the most promising endolysins with activity against *S. aureus* N305 in UHT-milk [38]. The latter was done by challenging 10^3^ CFU/mL *S. aureus* N305-spiked UHT-milk with *E. coli* lysates that contained the expressed endolysins. The selected top candidate (*i.e.*, ClyCHAPK_CWT-LST) combines the CHAP domain of PlyK with a SH3b CBD of which the origin is undefined. Interestingly, the activity of the selected engineered endolysin was equal to that of the positive control lysostaphin. Both were able to eradicate 10^3^ CFU/mL *S. aureus* N305 from UHT whole milk to undetectable levels in 3 h. Therefore, ClyCHAPK_CWT-LST was combined with lysostaphin to exploit synergy that was observed in broth and UHT whole milk (*i.e.*, Σ of 0.46 ± 0.04 and 0.38 ± 0.03, respectively). Different bovine mastitis-derived staphylococcal isolates, including 23 *S. aureus* isolates and one isolate of *Staphylococcus chromogenes* and *S. simulans*, were found susceptible with MBC values < 1.0 µM to ClyCHAPK_CWT-LST, lysostaphin and the synergistic combination. An exception to the latter was *S. chromogenes*, which yielded a MBC > 2.5 µM of the individual enzymes, but not for the synergistic combination that still had an MBC < 1.0 µM. To investigate the potential of ClyCHAPK_CWT-LST and the synergistic combination with lysostaphin, a kinetic TKA was performed in UHT-milk with 10^3^ and 10^6^ CFU/mL *S. aureus* N305. After 3 h, lysostaphin and ClyCHAPK_CWT-LST reduced bacterial concentrations by 2.64 and 2.37 log_10_ compared to the 10^3^ CFU/mL control at ± 1.0 µM, respectively. Their synergistic combination reduced the CFU count by 4.74 log_10_ after 3 h below the limit of detection in comparison with the 10^6^ CFU/mL control. However, when repeating the same assay in raw cow’s milk, ClyCHAPK_CWT-LST completely lost its activity which was retrieved by diluting the raw milk, clearly showing that raw milk components inhibit the activity of ClyCHAPK_CWT-LST. The authors repeated the screening a couple of years later but now used raw cow’s milk instead for UHT-milk and a novel engineered enzyme, named ClyM23LST(L)_SH3b2638, was hit-to-lead selected for staphylolytic activity [39]. This enzyme consists of the M23 EAD of lysostaphin and the C-terminal CBD of the staphylococcal phage 2638A endolysin [97]. Their selected ClyM23LST(L)_SH3b2638 had, however, still a few relevant limitations: (i) it did not perform better than the natural bacteriocin lysostaphin in raw cow’s milk (i.e., at least a twofold increase of the MBC against *S. aureus* N305 was present), (ii) it did not show synergy in UHT-milk with other endolysins that target different bonds in the staphylococcal peptidoglycan, and (iii) resistance against the M23 EAD of lysostaphin was mentioned. The reason for the latter is that lysostaphin’s M23 EAD hydrolyses the pentaglycine bridge in the staphylococcal peptidoglycan, which can be altered [98]. Interestingly, ClyM23LST(L)_SH3b2638 was further rationally engineered by fusion of the TAT peptide of HIV-1 to the C-terminus, which increased the construct’s capacity to kill *S. aureus* intracellularly in MAC-T. Cells treated with non-TAT engineered enzymes had approximately 10^5^ CFU/mL intracellular *S. aureus* N305 (i.e., approximately 5 CFU/cell), whereas this was reduced in boMECs that were treated with 1.0 µM of the TAT engineered variant during 3 h (i.e., ± 1.0 additional Δlog_10_). Noteworthy, the TAT fusion to ClyM23LST(L)_SH3b2638 decreased the antibacterial activity by ± 1.0 log_10_, observed with *S. aureus* N305 in buffer and diluted raw cow’s milk. This has also been reported for ClyK-L-PTD in comparison with ClyK-L, of which the former also contains the C-terminal TAT [43].

## Overall discussion and conclusion

Gram-positive bovine mastitis is a prevalent disease with significant economic implications for the dairy industry [99, 100]. While preventive measures such as hygiene practices, teat sealants, vaccination and probiotics have proven effective in controlling the disease, they do not result in a complete elimination [101, 102]. Consequently, there is a need for effective therapy. Antibiotics currently provide therapeutic relief, but their use is increasingly questioned, particularly antibiotics that are regarded critical for human health care [103, 104]. In this context, bacteriophage-derived endolysins have emerged as promising antimicrobials to either replace or complement existing treatments against Gram-positive bovine mastitis pathogens [3].

The first endolysin that came in focus of this review, PlyC, was discovered in 1957 by Krause [58]. After that, the endolysin technology gained increasing attention from the early 2000s on. This resulted in the rapid discovery of new and sometimes highly similar endolysins by different groups, as well as different nomenclatures [53]. While the initially discovered wild-type endolysins exhibited high promise under simplistic in vitro conditions, such as laboratory buffers or pasteurized milk, challenges persisted in raw milk and in vivo scenarios [35, 38]. For example, multiple studies reported a matrix inhibitory effect caused by raw milk, even though the endolysin initially displayed potent antimicrobial activity in pasteurized milk [31, 38]. Another study demonstrated synergy between two endolysins but reported that this synergistic action was lost in the murine mammary gland [35]. It is important to understand that endolysins are evolutionarily designed to release the bacteriophage from the infected host cell and cause ‘lysis-from-within’. Thus, their efficacy is not necessarily guaranteed when causing ‘lysis-from-without’, certainly not in the demanding conditions that they encounter in the infected mammary gland [6, 105]. This limitation has posed an initial drawback for the wild-type endolysins to achieve therapeutic breakthrough in veterinary medicine.

From 2012 onward, the engineering of endolysins has predominantly transpired, which is possible due to their modular structure [9]. This also enabled the addition of novel modules or peptides to expand their working spectrum beyond their conventional antimicrobial capabilities, such as incorporating CPPs to target mastitis pathogens within the bovine mammary epithelium [31, 39, 43]. In the meantime, endolysins also have been reported to target other virulence factors of Gram-positive bovine mastitis pathogens, such as eradicating biofilms and killing antibiotic resistant strains [31, 41, 106]. In addition, recent protein engineering methods have evolved from rational assembly to creating complex libraries, allowing for an extensive screening and hit-to-lead selection of candidates that display robust ‘lysis-from-without’ activity under the specific conditions encountered in the infected mammary gland [31, 93]. Indeed, the first library only consisted of a few dozen variants [55], which were later expanded to a few hundred variants [38, 40], and most recently, to tens of thousands of variants [31]. It must be emphasized that screening these libraries under the end-user conditions is crucial, as multiple groups have reported that screening in pasteurized milk does not guarantee activity in raw milk [31, 38, 39]. However, it became clear that these high-throughput protein engineering methods yield lead candidates that offer great therapeutic potential for the treatment of bovine mastitis [95].

The integration of endolysins as stand-alone products or in combination with non-critical antibiotics should be a focal point for future research. Isoxazolyl penicillins (e.g., oxacillin, cloxacillin) have been reported to be promising candidates for supplementation, potentially exhibiting synergy or a potentiating effect when used together with engineered endolysins [56, 95]. Considering that mastitis treatment typically occurs at milking with a 12-h interval, integration as a stand-alone product may be impractical due to the endolysin’s short half-life, although sufficient pharmacokinetic and -dynamic data about endolysins in a mastitis context are currently lacking [105]. Exploring their application as dry cow treatment in a slow-release formulation can also be proposed, as was recently reported for another veterinary application [107].

In conclusion, recent advances in modular protein engineering methods present novel opportunities for endolysins to simulate horizontal gene transfer in a high-throughput manner and select for lead candidates under the end user conditions. Identifying endolysin candidates capable of withstanding the demands of the lactating bovine mammary gland is now highly feasible. This review asserts that endolysin therapy, either as stand-alone or as a supplementation to antibiotics, is on the verge of a transformative breakthrough in veterinary medicine. Expectations are high that bacteriophage-derived endolysins will integrate into the antibiotic armamentarium over the next decade, revolutionizing the approach to combating bovine mastitis-causing streptococci and staphylococci. A promising era marked by innovative antimicrobial treatment strategies is about to begin, that will further shape the future of veterinary medicine.

## Data Availability

The datasets used and/or analysed during the current study are available from the corresponding author on reasonable request.

## Abbreviations

BME-UV1: Bovine mammary epithelial cell line BME-UV1
BoMECs: Bovine mammary epithelial cell lines
CBD: Cell wall-binding domain
CFU: Colony forming units
CHAP: Cysteine/Histidine-dependent Aminohydrolase/Peptidase
Cly: Chimeric endolysin
CPP: Cell penetrating peptide
EAD: Enzymatic active domain
GFP: Green fluorescent protein
H&E: Hematoxylin & eosin
HIV-1: Human immunodefficency virus 1
IL: Interleukin
Lys: Phage endolysin
MAC-T: Bovine mammary alveolar cell line stable transfected with SV-40 large T-antigen
MBC: Minimal bacteriocidic concentration
MBEC: Minimum biofilm eradicating concentration
MIC: Minimal inhibitory concentration
MRSA: Methicillin-resistant *Staphylococcus aureus*
OD_600nm_: Optical density measured at 600 nm
PBS: Phosphate buffered saline
pH: Potential of hydrogen
Phages: Bacteriophages
pI: Iso-electric point
Ply: Phage endolysin
PMN: Polymorphonuclear leukocytes
PS: Penn state bovine mammary epithelial cell line
SCC: Somatic cell count
TAT: Trans-activator of transcription
TKA: Time kill assay
TLR: Toll-like receptor
TNF: Tumor necrosis factor
TRA: Turbidity reduction assay
UHT: Ultra-high temperature treated
